# Psychosocial factors and the development of childhood overweight and obesity: a UK cohort study

**DOI:** 10.1038/s41390-025-04113-x

**Published:** 2025-05-13

**Authors:** I Gusti Ngurah Edi Putra, Michael Daly, Eric Robinson

**Affiliations:** 1 Department of Public Health, Policy and Systems, Institute of Population Health, University of Liverpool, Liverpool, UK; 2 Department of Psychology, Maynooth University, Kildare, Ireland; 3 Department of Psychology, Institute of Population Health, University of Liverpool, Liverpool, UK

## Abstract

**Background:**

We examined the role of psychosocial factors in overweight and obesity development.

**Methods:**

UK Millennium Cohort Study data of children with normal weight at baseline were analysed. Weight changes were determined from baselines at ages 11 (*n* = 7979) and 14 (*n* = 6906) to follow-up at age 17. Baseline individual psychosocial factors were combined into two distinct indexes (caregiver-reported child mental health, child-reported psychosocial well-being). Regression models examined the associations between baseline indexes and individual psychosocial factors and overweight and obesity development (vs. no development) and body mass index (BMI) z-score changes.

**Results:**

Worse child mental health, but not psychosocial well-being, at age 11 was associated with overweight and obesity development (OR = 1.14; 95% CI = 1.02, 1.27) and increased BMI z-scores (*β* = 0.08; 95% CI = 0.04, 0.12) to age 17. No psychosocial indexes at age 14 predicted the outcomes. Further analyses showed that child mental health at ages 11 vs. 14 was more likely to predict the outcomes. Based on individual factors, externalising symptoms and experiencing peer bullying at age 11 may be important contributors to overweight and obesity development.

**Conclusions:**

Poor child mental health at age 11 is associated with overweight and obesity development by age 17. Late childhood/early adolescence may be a sensitive period in which psychosocial factors predict body weight trajectories.

**Impact:**

- Worse psychosocial factors, particularly poor mental health, at ages 11, but not 14, were associated with overweight and obesity development and increased BMI z-scores by age 17.
- Late childhood/early adolescence may be a sensitive period for mental health in predicting future weight change.
- Future research will benefit from exploring this potential sensitive period and understanding potential mechanisms.

## Introduction

Across most developed countries, a large number of children and adolescents are now classed as living with overweight and obesity.^1^ Childhood obesity has been shown to track into adulthood and predict future disease burden.^2^ For example, a meta-analysis estimated adulthood obesity to be five times more likely among children with vs. without obesity.^3^ There is convincing evidence that family and socioeconomic factors predict development of child overweight and obesity. In higher-income countries, children of parents with obesity and children from lower socioeconomic households are at increased risk of developing overweight and obesity in childhood.^4^

Another important, but less studied and understood set of potential contributors to childhood overweight and obesity development risk are psychosocial factors, such as poorer child mental health, low self-esteem, and negative social relationships (e.g. social support, experience of bullying).^5^ The majority of studies linking psychosocial factors to childhood overweight and/or obesity have been cross-sectional.^6,7^ Because living with obesity may negatively impact psychosocial factors, such studies do not provide evidence on whether psychosocial factors increase prospective risk of developing overweight and obesity.

A smaller, more recent body of research has started to examine if individual psychosocial factors predict child weight gain in isolation. Initial evidence suggests that experiencing peer victimisation may predict weight gain in childhood.^8^ In addition, studies have examined other psychosocial predictors, such as mental health defined into internalising and externalising symptoms, separately.^9–11^ As many psychosocial factors are likely to be inter-related (e.g. experiencing peer victimisation and worse mental health are comorbid),^12^ it is unclear if previously identified psychosocial factors are independent predictors of weight gain and if they contribute to an overall psychosocial profile of risk with a strong prognostic ability in predicting the development of overweight and obesity. The latter proposition is consistent with contemporary research, which suggests that there may be underlying latent dimensions (or general factor) relating to psychosocial well-being or mental health, which can be considered general risk factors in the development of mental or physical health problems.^13^ Therefore, further investigation is needed to determine whether a set of psychosocial factors that are conceptually related should be combined, as they may collectively provide a more comprehensive understanding of overall profiles of mental health or psychosocial well-being that predict overweight and obesity development.

In the present research, we examined the prognostic value of different psychosocial factors in explaining risk of developing childhood overweight and obesity. To achieve this, we used nationally representative longitudinal cohort data of children living in the UK, the Millennium Cohort Study (MCS), for which a wide range of available psychosocial measures may be associated with weight or weight changes based on previous studies, including internalising and externalising symptoms,^10,11^ depressive symptoms,^14^ peer victimisation,^8^ appearance satisfaction,^15^ life satisfaction,^16^ and self-esteem.^15^ These psychosocial factors may be associated with the development of overweight and obesity through biological and/or behavioural mechanisms.^17–20^ For example, a multitude of psychosocial factors have been shown to predict unhealthy eating patterns (e.g. emotional eating, consumption of high-calorie foods) and secretion of the hormone cortisol, which together can contribute to an accumulation of fat in visceral adipose tissues.^21,22^

Using MCS, we first tested how individual psychosocial factors were conceptually related to each other using factor analysis, and then evaluated the explanatory power of overall profiles of child mental health or psychosocial well-being in predicting the development of overweight or obesity. To be consistent with previous research, we also examined individual psychosocial factors in isolation. This approach allows us to better understand if some individual variables that constituted overall profiles were particularly important in explaining the development of overweight and obesity. There is limited evidence of significant comorbidity between heavier body weight and psychosocial measures during early childhood, and the most extensive range of psychosocial measures available in MCS is collected at age 11 onward.^23,24^ In the present research, we therefore focused on whether psychosocial factors predict the development of overweight and obesity from age 11 through to age 17.

## Methods

### Study design and data

Data for this study came from a birth cohort of children living in the UK, the MCS. Children or cohort members were recruited using a probability sampling method stratified by country, disadvantaged circumstances, and ethnicity.^23^ We used Sweeps 5 (age 11; 6-year follow-up) and 6 (age 14; 3-year follow-up) as different study baselines due to a diverse range of psychosocial factors available and examined the role of baseline psychosocial factors in predicting the development of overweight and obesity at Sweep 7 (age 17). For the analysis, cohort members with underweight at  both baseline and follow-up and with overweight and obesity at baseline were excluded. Sample sizes were *n* = 7979 and *n* = 6906 children with normal weight at baselines (ages 11 and 14, respectively). MCS received ethics approval from the National Health Service Research Ethics Committee and the National Research Ethics Service. Informed consent was obtained from participating caregivers (cohort members at age ≤14) and directly from cohort members at age 17.

### Variables

#### Weight status

Body mass index (BMI) in kg/m^2^ was calculated based on measurements of weight and height collected by a trained interviewer and then transformed into BMI z-scores. BMI category at baseline and follow-up was then defined using the British 1990 child growth reference population (UK90)^24–26^ (see Supplementary Materials). As we aimed to assess the development of overweight and obesity at follow-up, children living with overweight and obesity at baseline were excluded.

#### Psychosocial factors

We followed our previous study^24^ to examine cohort member- or caregiver-reported psychosocial factors in this study (see Table 1). Previous work using MCS datasets (e.g.^15,24,27,28^) informed how these psychosocial factors were quantified (see Supplementary Materials). We converted all psychosocial factors into z-scores to enable comparison. We used exploratory factor analysis (EFA) to identify the underlying factor structure of psychosocial factors as they may be conceptually related. We included psychosocial factors consistently available across baselines (*n* = 7, see Table 1) in EFA and found two distinct factors: (1) child-reported psychosocial well-being and (2) caregiver-reported child mental health (see Table 1 and Supplementary Materials) (e.g. as in ref. ^24^). Related standardised psychosocial factors were averaged and re-standardised to create an index (mean = 0; standard deviation (SD) = 1). A higher index indicated worse mental health and psychosocial well-being (see Supplementary Materials).

**Table 1:** Measured psychosocial variables in the study.

Measures	Baseline measurements		Indexes of psychosocial factors
	Age 11	Age 14	
Appearance satisfaction	Child reported	Child reported	Child-reported psychosocial well-being
Callous/unemotional traits	Child reported		*NA*
Depressive symptoms	Child reported	Child reported	Child-reported psychosocial well-being
Externalising symptoms	Caregiver reported	Caregiver reported	Caregiver-reported child mental health
Internalising symptoms	Caregiver reported	Caregiver reported	Caregiver-reported child mental health
Life satisfaction	Child reported	Child reported	Child-reported psychosocial well-being
Online bullying victimisation		Child reported	*NA*
Peer bullying victimisation	Child reported	Child reported	Child-reported psychosocial well-being
Self-esteem	Child reported	Child reported	Child-reported psychosocial well-being
Social support		Child reported	*NA*

We used exploratory factor analysis (EFA) with varimax rotation to identify separate or distinct psychosocial factors (as in our previous study^24^). Full EFA information and analyses are in Supplementary Materials.

*NA* not applicable, excluded from the  EFA approach as unavailable in multiple waves.

#### Covariates

We included covariates in the analysis, including sex, family structure, ethnicity, and three socioeconomic status (SES) measures (caregiver education, occupation, and household income; see Supplementary Materials) (as in refs. ^24,29^). We also controlled for pubertal status reported at baseline as pubertal status may confound associations (i.e. pubertal status is associated with both weight change^30^ and psychosocial factors^31^). In addition, changes in pubertal status from ages 11 to 14 were controlled for analysis of age 11 as baseline (see Supplementary Materials).

### Data analysis

#### Main analyses

We pre-registered our analytical approach at 10.17605/OSF.IO/QMHXW. We used binary logistic regression to examine the association between the indexes of psychosocial factors (caregiver-reported child mental health and child-reported psychosocial health) at baselines (ages 11 or 14) and the development of overweight and obesity (vs. no development) at follow-up (age 17). As EFA showed that these two indexes were distinct factors, both were fitted together in the same regression model, controlling for covariates. We presented the findings as odds ratio (OR), 95% confidence intervals (CI) of OR and *p* value.

We also estimated the extent to which psychosocial factors were associated with continuous changes in BMI z-scores between baseline and follow-up. Changes in BMI z-scores were presented as residualised change scores, calculated by regressing follow-up BMI z-scores against baseline values.^24,32^ We used linear regression to examine the links between the indexes of psychosocial factors and BMI z-score changes. Regression coefficient (*β*), 95% CI of *β*, and *p* value were presented.

To address potential selection bias due to missing observations, we used multiple imputation by chained equations (MICE). Data were also converted for complex survey design analysis following multiple imputation (see Supplementary Materials). We set the significance level at *p* < 0.05 for the main analyses and we used the Benjamini-Hochberg adjustment method^33^ for the following analyses to control the false discovery rate.

#### Sensitivity/additional analyses

We adjusted baseline BMI z-scores for regression models predicting the main categorical outcome (development vs. no development of overweight and obesity). Separate regression models were developed for individual psychosocial factors (as opposed to the two indexes in main analyses) in predicting the outcomes (development vs. no;  BMI z-score changes). We examined whether the associations between indexes of psychosocial factors and the outcomes were moderated by SES. We combined the three SES measures to develop a composite index of SES following a previous study.^34^ All the three SES indicators were reverse-coded to indicate more social disadvantage by a higher score, z-score transformed, and then averaged. We re-standardised the average values to create an SES index. Two-way interaction terms between psychosocial factor indexes and an SES index were included in the regression models in predicting the outcomes.

## Results

### Characteristics of participants

Table 2 presents sociodemographic characteristics of participants with normal weight at baseline. Similar proportions between males and females were included. The majority of participants were White (>80%) and lived with two caregivers (>70%). Most of the participants were from families with higher SES. Compared to excluded participants (i.e. living with overweight and obesity at baseline), participants in our study (i.e. living with normal weight at baseline) were more likely to be from families with higher SES (higher levels of education, occupation, and income) (*findings are not presented)*.

**Table 2:** Characteristics of participants with normal weight at baseline.

Variables	Age 11		Age 14	
	*n* = 7979	%	*n* = 6906	%
Sex				
Male	3938	50.55	3533	53.26
Female	4041	49.45	3373	46.74
Ethnicity				
White	6708	85.75	5493	80.86
Non-White	1270	14.23	1396	19.04
Missing	1	0.01	17	0.27
Family structure				
Two-caregiver family	6234	74.79	5368	72.30
One-caregiver family	1745	25.21	1537	27.67
Missing	0	0	1	0.03
Caregiver education				
No qualification	428	7.05	331	7.12
Overseas qualification	162	1.95	128	1.93
NVQ level 1	372	5.56	286	5.44
NVQ level 2	1557	21.74	1153	20.57
NVQ level 3	1182	14.91	975	14.41
NVQ level 4	2905	34.22	2640	34.13
NVQ level 5	1276	14.00	1377	15.96
Missing	43	0.57	16	0.44
Caregiver occupation				
Unemployed	1498	21.22	975	17.29
Semi-routine and routine	1030	13.53	932	16.24
Technical, low supervisory	313	3.93	266	4.09
Small employers, self-employed	703	8.57	622	9.53
Intermediate	960	12.09	875	12.10
Professional, managerial	3449	40.31	3236	40.75
Missing	26	0.35	0	0
Household income				
Quartile 1 (bottom)	1433	19.47	1010	17.05
Quartile 2	1420	18.43	1063	18.61
Quartile 3	1635	19.06	1375	19.60
Quartile 4	1743	20.45	1676	21.58
Quartile 5 (top)	1748	22.59	1776	23.07
Missing	0	0	6	0.09
Development of overweight and obesity				
No	4694	56.10	4738	64.94
Yes	879	10.80	653	9.25
Missing	2406	33.10	1515	25.81

For age 11 as the baseline, the weighted proportions of participants with and without development of overweight and obesity at follow-up were 16.14% and 83.86%, respectively, when only participants without missing information on the outcome were included in the calculation.

For age 14 as the baseline, the weighted proportions of participants with and without development of overweight and obesity at follow-up were 12.47% and 87.53%, respectively, when only participants without missing information on the outcome were included in the calculation.

*n* sample size, *%* weighted percentage, *NVQ* National Vocational Qualification.

### Indexes of psychosocial factors and the development of overweight and obesity

Table 3 shows that caregiver-reported child mental health, but not child-reported psychosocial well-being at age 11, was associated with the development of overweight and obesity at age 17. An increase (worsening) in caregiver-reported child mental health by one SD was associated with increased likelihood of development of overweight and obesity (vs. no) by 14% (OR = 1.14; 95% CI = 1.02, 1.27). The magnitude of this association remained the same after controlling for baseline BMI z-scores at *p* = 0.02 (not statistically significant after correcting for multiple comparisons) (Table S1). Worse caregiver-reported child mental health was also associated with increased BMI z-scores between ages 11 and 17 (*β* = 0.08; 95% CI = 0.04, 0.12) (Table 3). When indexes of psychosocial factors were fitted at age 14, neither was associated with the outcomes (development vs. no; residualised change BMI z-scores) at age 17 (Table 3). Additional analyses indicated no evidence of the association between indexes of psychosocial factors and the outcomes moderated by SES (Table S2).

**Table 3:** Associations between indexes of psychosocial factors and the development of overweight and obesity to a follow-up of age 17.

Psychosocial factors	Baseline: Age 11 (*n* = 7979)						Baseline: Age 14 (*n* = 6906)					
	Development (vs. no) by age 17			Residualised change BMI z-scores from age 11 to 17			Development vs. no by age 17			Residualised change BMI z-scores from age 14 to 17		
	OR	95% CI	*p* value	*β*	95% CI	*p* value	OR	95% CI	*p* value	*β*	95% CI	*p* value
Child-reported psychosocial well-being	1.04	0.95, 1.15	0.378	0.00	−0.03, 0.03	0.935	1.11	1.00, 1.23	0.063	−0.01	−0.03, 0.02	0.620
Caregiver-reported child mental health	1.14	1.02, 1.27	0.018	0.08	0.04, 0.12	<0.001	1.01	0.91, 1.12	0.826	−0.00	−0.03, 0.03	0.970

Indexes of psychosocial factors were presented in z-scores (mean = 0; SD = 1).

Both indexes were analysed in the same regression model, adjusting for sociodemographic covariates and pubertal status.

*OR* odds ratio, *β* regression coefficient, *CI* confidence intervals.

We also conducted non-pre-registered exploratory analyses to understand whether more pronounced association between caregiver-reported child mental health at age 11, but not age 14, and the outcomes at age 17 due to a longer follow-up period (6 years vs. 3 years). We examined whether caregiver-reported child mental health at age 11 predicted the development of overweight and obesity for a shorter follow-up period (3 years) by using age 14 as the follow-up. Findings indicated caregiver-reported child mental health at age 11 was associated with the development of overweight and obesity (OR = 1.25; 95% CI = 1.11, 1.41) and increased BMI z-score (*β* = 0.07; 95% CI = 0.05, 0.09) by age 14 (Table S3).

Caregiver-reported child mental health at age 11 was consistently associated with the development of overweight and obesity and increased BMI z-scores, irrespective of follow-up period, suggesting this index may be more important at ages 11 than 14 in predicting the development of unhealthy weight. We decided to formally examine differences in predictive ability based age at baseline. We added two-way interaction terms between study baselines (ages 11 vs. 14) and caregiver-reported child mental health into regression models in predicting the outcomes at age 17. Findings indicated statistically significant interactions, suggesting a larger effect of caregiver-reported child mental health at age 11 vs. 14 in predicting residualised change BMI z-scores at age 17 (Table S4).

### Individual psychosocial factors and the development of overweight and obesity

Findings from examining individual psychosocial factors were supportive of the main analysis in which components of caregiver-reported child mental health, such as internalising and externalising symptoms at age 11 (not age 14) were associated with increased BMI z-scores at age 17 and externalising symptoms were also associated with the development of overweight and obesity (vs. no) (Table 4). Internalising and externalising symptoms at age 11 were also predictive of both outcomes (the development of overweight and obesity, increased BMI z-scores) for a shorter follow-up period at age 14 (Table S5). Both symptoms also had larger effects when assessed at ages 11 vs. 14 in predicting BMI z-score changes at age 17 (Table S6). Even though the index of child-reported psychosocial well-being was not associated with the outcomes, experiencing peer bullying at age 11, but not at age 14, was associated with increased BMI z-score at age 17 (Table 4). Peer-bullying experience at age 11 also predicted an increase in BMI z-scores at age 14 (Table S5), and had larger predictive ability compared to at age 14 for BMI z-score changes at age 17 (Table S6).

**Table 4:** Associations between individual psychosocial factors and the development of overweight and obesity to a follow-up of age 17.

Psychosocial factors	Baseline: Age 11 (*n* = 7979)						Baseline: Age 14 (*n* = 6906)					
	Development (vs. no) by age 17			Residualised change BMI z-scores from age 11 to 17			Development (vs. no) by age 17			Residualised change BMI z-scores from age 14 to 17		
	OR	95% CI	*p* value	*β*	95% CI	*p* value	OR	95% CI	*p* value	*β*	95% CI	*p* value
Appearance satisfaction	0.92	0.84, 1.00	0.050	−0.01	−0.04, 0.02	0.426	0.87	0.78, 0.96	0.008	0.00	−0.03, 0.03	0.920
Callous/unemotional traits	0.99	0.89, 1.10	0.904	0.01	−0.02, 0.04	0.485	NA			NA		
Depressive symptoms	1.07	0.98, 1.18	0.128	0.03	−0.00, 0.05	0.077	1.09	0.97, 1.22	0.154	0.00	−0.02, 0.03	0.732
Externalising symptoms	1.19	1.08, 1.31	0.001*	0.09	0.06, 0.13	<0.001*	1.04	0.93, 1.17	0.486	0.01	−0.02, 0.04	0.477
Internalising symptoms	1.09	1.00, 1.20	0.060	0.05	0.01, 0.08	0.007*	1.02	0.92, 1.13	0.725	−0.02	−0.05, 0.01	0.289
Life satisfaction	1.04	0.95, 1.13	0.417	0.01	-0.03, 0.04	0.758	0.93	0.84, 1.03	0.162	0.01	−0.02, 0.03	0.547
Online bullying	NA			NA			1.12	1.02, 1.24	0.017	0.01	−0.01, 0.04	0.264
Peer bullying	1.14	1.04, 1.26	0.006	0.06	0.03, 0.09	<0.001*	0.97	0.88, 1.08	0.602	−0.01	−0.04, 0.01	0.322
Self-esteem	0.93	0.86, 1.01	0.098	−0.01	−0.04, 0.02	0.378	0.88	0.80, 0.98	0.015	0.00	−0.02, 0.03	0.862
Social support	NA			NA			0.99	0.88, 1.10	0.800	0.02	−0.01, 0.04	0.156

All individual psychosocial factors were presented in z-scores (mean = 0; SD = 1).

Separate regression models were developed for each individual psychosocial factor, adjusting for sociodemographic covariates and pubertal status.

*OR* odds ratio, *β* regression coefficient, CI confidence intervals, *NA* not applicable (i.e. psychosocial factor was not available).

^*^The *p* value is statistically significant based on the Benjamini-Hochberg correction method (see Tables S9 and S10).

Having confirmed three individual psychosocial factors (internalising, externalising symptoms, peer bullying) at age 11 had strong associations with weight outcomes by ages 14 and 17, we conducted exploratory analyses (non-preregistered) to examine the independent contribution by fitting these three factors in the same regression model (as opposed to separate models). We found that externalising symptoms and peer bullying at age 11 were independently associated with BMI z-score changes at age 17 (Table S7). Externalising symptoms at age 11 were also independently associated with BMI z-score changes at age 14 (Table S8).

## Discussion

Using nationally representative data of UK children, we found that studied psychosocial measures related to one of two overarching factors: caregiver-reported child mental health (characterised by internalising and externalising symptoms) and child-reported psychosocial well-being (characterised by self-esteem, life satisfaction, appearance satisfaction and experienced victimisation). In primary analyses, overall poorer child mental health reported by caregivers at age 11, but not 14, was a statistically significant and independent prospective risk factor of overweight and obesity development and/or increased BMI z-scores by ages 14 and 17. Children with worse mental health at age 11 were 14% more likely to have developed overweight and obesity by age 17. Conversely, overall child-reported psychosocial well-being at age 11 years was not an independent significant prospective predictor of weight outcomes by age 14 or 17. There was no evidence that the prospective relationships between caregiver-reported child mental health, child-reported psychosocial well-being, and the development of overweight and obesity were moderated by family-level SES.

There was consistent evidence of significant prospective risk when child mental health was measured at age 11 years, but no evidence of significant prospective risk when measured at age 14 years. These findings may be interpreted as providing evidence that late childhood/early adolescence is a sensitive period in which child mental health increases risk of weight gain, compared to later in adolescence. For some children, early adolescence will involve social changes and be a stressful period in which the onset of mental health difficulties occurs.^35,36^ Changes in social circumstances, such as the transition from primary to secondary school, may contribute to stress and mental health difficulties in UK children during this period (e.g. age 11).^37,38^ It is therefore feasible that worse mental health during this period may result in the development of unhealthy coping strategies (e.g. unhealthy diet, emotional eating)^19,20^ which become engrained over time and contribute to increased weight gain.^39^ Conversely, by age 14, these factors may be less pertinent. As findings regarding age were largely unexpected and our interpretations are speculative, further research examining if late childhood or early adolescence is a sensitive period for mental health-related risk of weight gain, and explanatory mechanisms is now needed. Future research will benefit from adopting a life-course perspective that incorporates repeated data on psychosocial factors and weight status across developmental stages from childhood to adulthood. This would clarify how associations may change over time, identifying critical periods for intervention.

A strength of this present study was that we were able to examine an extensive range of psychosocial measures. Previous research has examined relationships between psychosocial factors and child adiposity by studying a limited number of psychosocial factors independently. For example, internalising and externalising symptoms are known to be interrelated, but their associations with child weight gain have at times been studied in isolation,^9–11^ which prevents making conclusions about their potential independent contributions. Analyses in the present study indicated that the extensive range of measures examined was characterised by and largely related to a two-factor solution: caregiver-reported child mental health and child-reported psychosocial well-being. In primary analyses, we examined how these two overarching factors related to prospective risk of child overweight and obesity development.

Our primary findings suggest that presented mental health difficulties (caregiver reporting internalising and externalising symptoms) may be more important for overweight and obesity development risk than how overall children feel about themselves and their social world (psychosocial well-being index). This may be because coping strategies involving unhealthy eating are more pronounced in children experiencing mental health difficulties. A previous study showed that mental health difficulties, defined by the same tool (i.e. subscales of the Strength and Difficulties Questionnaire (SDQ) —see Supplementary Materials) were associated with higher intakes of sweet and fatty foods in children, but such relationships were not observed for emotion-related measures not necessarily indicative of experiencing significant mental health problems (i.e. feelings of anxiety, sadness, anger, happiness).^20^ While our findings showed the potential role of mental health difficulties in the development of overweight and obesity, current literature on the bidirectional nature of distress and weight^40^ may indicate a contribution of subsequent psychological burden of living with overweight and obesity in maintaining weight gain and explaining why weight loss or overweight and obesity reversal is difficult.

The index approach we used in this study may serve as a potential strength as the index combined conceptually related psychosocial factors, and our findings suggest that at a global level, child mental health may be particularly important in explaining overweight and obesity risk, as opposed to measures of psychosocial well-being. In analyses limited to individual variables, we found strong evidence that internalising, externalising symptoms, and peer bullying at age 11 were associated with weight outcomes by ages 14 and 17 when assessed separately. When these three psychosocial factors were examined in concert, only externalising symptoms and peer bullying were observed to have an independent contribution. Internalising symptoms were no longer associated with the outcomes when externalising symptoms were included in the model, indicating both were conceptually related and that externalising symptoms may be the primary driver of the mental health and overweight and obesity risk development. Our findings do suggest that overall profiles of mental health or psychosocial well-being do have predictive validity in explaining the development of overweight and obesity and when individual measures are numerous and/or highly correlated, taking an overall profile or ‘index’ approach may be useful in reducing number of statistical tests and avoiding collinearity issues. Nonetheless, whilst overall psychosocial well-being did not tend to predict overweight and obesity development independent to child mental health, one individual psychosocial measure (peer bullying) was uniquely associated with weight gain risk.

Stronger associations for some individual psychosocial factors examined in concert, such as externalising symptoms (defined by conduct problems and hyperactivity in SDQ), align with findings from past work. For example, conduct disorder symptoms are prospectively linked to increased BMI and/or obesity development,^41,42^, partly due to physical inactivity.^42^ Similarly, a meta-analysis reported a strong association between attention-deficit/hyperactivity disorder and overweight or obesity development,^43^ that may be explained by unhealthy eating patterns and sedentary behaviours.^44^ Our findings on peer-bullying victimisation and the development of overweight and obesity align with other studies,^8,45,46^ and unhealthy eating behaviours (e.g. eating to cope with negative emotions) may in part explain this relationship.^47,48^ In addition to behavioural coping mechanisms, biological responses, such as elevated cortisol levels and hypothalamic-pituitary-adrenocortical axis activation, may explain weight gain following exposure to chronic stress.^18,21,49,50^ Further research is now warranted to explore whether other unmeasured psychosocial factors explain weight gain in adolescence and their potential mechanisms.

 Our analyses focused on ages 11 to 17 years old due to the availability of an extensive range of psychosocial measures in MCS. Further research would therefore benefit from examining whether similar findings are observed during early childhood. Although prior to age 7, there is minimal evidence of prospective relations between internalising symptoms and weight gain in UK and US children,^9,11^, it may be the case that other mental health and psychosocial well-being measures are associated with significant obesity risk earlier in life.^10^

Strengths of the present research included the use of nationally representative cohort data and repeated objective measurements of child weight. We used recognised BMI z-score cut-offs to define cases of child overweight and obesity. BMI z-scores account for child sex and age, and are widely used to characterise child adiposity, but can lack specificity at very high levels of adiposity and do not account for body composition.^51^ Other markers of child adiposity (e.g. body fat percentage, waist-to-height ratio) may better characterise child adiposity,^52^ and predicting changes in these markers from psychosocial factors is now warranted. Although the majority of psychosocial measures examined were from validated and widely used questionnaires, differences in findings observed between caregiver-reported child mental health vs. child-reported psychosocial well-being measures may be in part due to differences in respondents. The sample examined was was predominantly White, which limits the generalisability of results to other ethnicities. In addition, data on medication use was not available, and as medication use may be related to weight gain, this is a limitation of the study.

Another strength of our analysis that we controlled for pubertal status which may confound the association between psychosocial factors and overweight and obesity development due to its links to psychosocial factors^31^ and weight status.^30^ Future research may benefit from exploring whether psychosocial factors are associated with earlier pubertal onset and testing the role of pubertal status in moderating the relationship between psychosocial factors and the development of excess weight.

## Conclusions

Psychosocial factors at age 11 were associated with the development of overweight and obesity by ages 14 and 17. Late childhood/early adolescence (age 11) may be a sensitive period in which some psychosocial factors shape weight trajectories and increase risk of developing overweight and obesity. Intervention to improve mental health during late childhood may contribute to decrease population-level overweight and obesity risk into mid and late adolescence.

## Supplementary information

Supplementary materials

## Data Availability

Deidentified individual participant data (including data dictionaries) are available at 10.5255/UKDA-Series-2000031 with the permission of the UK Data Service.
