# Supplementary material for: Psychosocial factors and the development of childhood overweight and obesity: a UK cohort study
Source: Pediatr Res. 2025 May 13;98(6):2132–8. doi: 10.1038/s41390-025-04113-x (PMC12811144; doi:10.1038/s41390-025-04113-x)
Supplement: Supplementary file 1 — Supplementary materials [file 41390_2025_4113_MOESM1_ESM.pdf]

Supplementary information

Weight status

Trained interviewers examined cohort members' weight and height using standardised instruments. Body mass index (BMI) was calculated in kg/m^2. Following a previous study,[1] we excluded participants whose BMI < 10 or > 50 kg/m^2. The British 1990 child growth reference population (UK90)[2] was used to transform BMI into BMI z-scores in STATA (command: zanthro; see [3]), accounting for age and sex at the time of data collection (at ages 11, 14, 17). A derived variable of the UK90-reference BMI category was available for ages 14 and 17 in the datasets. To create the same BMI classification for age 11, we first converted BMI z-scores into percentiles and then used the UK90 reference percentile cut-offs.[4,5]

Psychosocial factors

Internalising and externalising symptoms

The Strengths and Difficulties Questionnaire (SDQ)[6] was administered to caregivers when children aged 11 and 14 years to assess child mental health difficulties (internalising and externalising symptoms). This questionnaire consists of five subscales (peer problems, emotional symptoms, hyperactivity, conduct problems, and prosocial behaviour) with five items for each subscale. A total of 25 items from the SDQ (e.g., "Many worries, often seems worried", "Often fights with other children or bullies them") were rated on a 3-point Likert scale. Responses, such as "not true", "somewhat true", and "certainly true" were coded as 0, 1, and 2, respectively for items of peer problems, emotional symptoms, hyperactivity, and conduct problems, but reverse coded for prosociality. A higher score on a scale of 0 to 10 for a total score indicated more mental health difficulties for four deficit-focused subscales, but favourable conditions for prosocial behaviour. Internalising symptoms were quantified by totalling the scores of peer problems and emotional symptoms, whereas the other two problem subscales, hyperactivity and conduct problems, constituted externalising symptoms (e.g., as in [7,8]). Each symptom had a final score ranging from 0 to 20 with a higher score indicating greater mental health problems or difficulties.

Self-esteem

A version of the Rosenberg Self-Esteem Scale[9] shortened to consist of five items was included in the young people self-completion module (e.g., "On the whole, I am satisfied with myself", "I feel that I have a number of good qualities") at ages 11 and 14. Responses on a 4-point Likert scale (from "strongly disagree" = 1 to "strongly agree" = 4) were added together, resulting in a total score on a scale of 1 to 20 (e.g., as in [8,10,11]). A higher score was indicative of higher self-esteem.

Callous/unemotional traits

Cohort members at age 11 completed a shortened four-item (e.g., "I feel bad or guilty when I have done something wrong", "I do not show my emotions to others") version of the Inventory of Callous-Unemotional Traits.[12] This scale consists of three subscales, namely uncaring (two items), callousness (one item), and unemotional (one item). Responses on a 4-point Likert scale (from "not at all true" = 0 to "definitely true" = 3) for each item were summed to create a summary score ranging from 0 to 12 (e.g., as in [8]). Greater callous or unemotional traits were indicated by a higher score.

Depressive symptoms

At age 11, we defined depressive symptoms based on three 5-point Likert-scale items (from "never" = 1 to "almost always" = 5) self-completed by cohort members: "In the last four weeks, how often did you feel sad?", "get worried about what would happen to you?", and "feel afraid or scared?" (e.g., as in [8,13]). All the responses were totalled to generate a summary score ranging from 1 to 15, with worse depressive symptoms indicated by a higher score. Good internal consistency was observed for this three-item scale of depressive symptoms.[13]

Depressive symptoms of 14-year-old participants were examined using the Short Mood and Feelings Questionnaire (SMFQ).[14] Responses to 13 items on a 3-point Likert scale (from "not true" = 1 to "true" = 3) (e.g., "I felt miserable or unhappy", "I didn't enjoy anything at all") were summed, resulting in a total score on a scale of 1 to 39. Greater depressive symptoms (negative moods and feelings) were indicated by a higher score (e.g., as in [8,13]).

Life satisfaction

We followed past studies using MCS[8,10,15] to define life satisfaction at ages 11 and 14 based on five 7-point Likert-scale items on the extent to which cohort members felt happy in different aspects in their life (e.g., family, friends, life as a whole). Their responses from "not at all happy" = 1 to "completely happy" = 7 for each item were added together to create a summary score ranging from 1 to 35. A higher score was indicative of better life satisfaction.

Appearance satisfaction

Following past work,[1,8,11,13] appearance satisfaction at ages 11 and 14 was determined using an item on the extent to which cohort members were happy about their appearance ("How do you feel about the way you look?"; with responses ranging from "not at all happy" = 1 to "completely happy" = 7). Greater appearance satisfaction was indicated by a higher summary score on a scale of 1 to 7.

Bullying victimisation

At age 11, cohort members completed a 6-point Likert-scale question on their bullying victimisation experience: "How often do other children hurt you or pick on you on purpose?"; "most days" = 1 to "never" = 6). An additional item with the same options for responses on online bullying victimisation experience was asked at age 14: "How often have other children sent you unwanted or nasty emails, texts or messages or posted something nasty about you on a website?". We re-coded the responses above to make a higher score indicate more frequent bullying experiences (e.g., as in [8,16]). Compared to our pre-registered analysis protocol (https://doi.org/10.17605/OSF.IO/QMHXW), bullying experience from siblings collected at ages 11 and 14 was not included. This was because 7% of the participants reported having no siblings, which resulted in more cohort members with missing information. Multiple imputations may not be considered appropriate to address these missing observations (see the 'Data analysis' section).

Social support

The Young Person Social Provisions Scale[17] was used to quantify perceived social support. Cohort members responded to three 3-point Likert-scale items (from "not true at all" = 1 to "very true" = 3) regarding their current interpersonal relationships (e.g., "I have family and friends who help me feel safe, secure and happy", "There is someone I trust whom I would turn to for advice if I were

having problems”). A total score on a scale of 1 to 9 was produced by adding together the responses across all the items. A higher score was indicative of stronger social support (e.g., as in [8,16]).

The development of indexes of psychosocial factors

We used exploratory factor analysis (EFA)[18] to better understand the underlying factor structure of psychosocial factors as they may be correlated and conceptually related. Only psychosocial factors collected at both ages 11 and 14 were analysed for EFA (n = 7), as this would allow comparisons on whether the time of psychosocial factors being assessed is more important in predicting the development of overweight and obesity. Two factors from EFA with Eigenvalue > 1.0 were retained. Varimax rotation was then used to identify separate psychosocial factors contributing to each identified distinct factor.[19] We found that these seven psychosocial factors were best characterised into two distinct factors: caregiver-reported child mental health defined by internalising and externalising symptoms, and child-reported psychosocial well-being, constituted by the remaining psychosocial factors (appearance satisfaction, depressive symptoms, life satisfaction, peer bullying, self-esteem) (e.g., as in [8]).

To develop an index for each distinct factor, positive psychosocial factors were re-coded to make a higher score indicate a more negative outcome (appearance satisfaction, life satisfaction, self-esteem). All the psychosocial factors were then z-score standardised. For each factor, corresponding standardised individual psychosocial factors (e.g., internalising and externalising symptoms for caregiver-reported child mental health) were averaged and re-standardised to construct a composite index (mean = 0; standard deviation (SD) = 1). To test convergent validity, correlations between individual psychosocial factors and the indexes were tested using Spearman’s rank correlation.[20] We found stronger correlations between indexes and their related psychosocial factors. Acceptable internal consistency was observed with Cronbach’s Alpha of 0.63 and 0.80 for indexes of caregiver-reported child mental health and child-reported psychosocial well-being, respectively.

Item loadings for psychosocial factors

Psychosocial factors	Item loadings	
	Factor 1	Factor 2
Appearance satisfaction	**0.843**	-0.025
Depressive symptoms	**0.785**	0.187
Externalising symptoms	0.036	**0.844**
Internalising symptoms	0.143	**0.823**
Life satisfaction	**0.773**	0.194
Peer bullying	**0.434**	0.272
Self-esteem	**0.808**	0.074

Correlation between individual psychosocial factors and indexes

Psychosocial factors	Correlation coefficients	
	Index of child-reported psychosocial well-being	Index of caregiver-reported child mental health
Appearance satisfaction	**0.794*****	0.108***
Depressive symptoms	**0.760*****	0.224***
Externalising symptoms	0.176***	**0.815*****
Internalising symptoms	0.240***	**0.835*****
Life satisfaction	**0.811*****	0.267***
Peer bullying	**0.540*****	0.131***
Self-esteem	**0.765*****	0.200***

***p < 0.001

Covariates

Socioeconomic status (SES) measures that were controlled in this study consisted of caregiver education, occupation, and household income (e.g., as in [8]). The highest vocational or academic qualification was grouped into the National Vocational Qualification (NVQ) levels 1 to 5, and additional groups were created for no qualification and overseas qualifications.[21] Occupational groups were determined following the National Statistics Socio-economic Classification, consisting of 1) semi-routine and routine occupations; 2) technical and lower supervisory; 3) small employers and self-employed; 4) intermediate; 5) managerial, administrative, and professional; and an additional category for 6) unemployed was added.[22] We selected the highest social group (education, occupation) for cohort members who lived with two caregivers (main caregiver and partner) from different groups.[21] We used a derived variable of equivalised weekly household income, taking into account the number of people living in the same household, and then categorised it into quintiles.[11]

We controlled pubertal status that was not originally included in our pre-registered analysis approach. Pubertal status may confound the association between psychosocial factors and the outcomes (the development of overweight and obesity, residualised BMI change z-scores). Following previous work,[23] pubertal status was defined by having first menstruation in females and voice deepening in males. Baseline pubertal status was controlled in the analyses predicting the outcomes at age 17. In addition, we created a variable indicating changes in pubertal status from ages 11 to 14, denoting "yes" if puberty occurred at age 14 but not at age 11. This variable, along with baseline pubertal status at age 11, was controlled in regression models with age 11 as the baseline (e.g., as in [8]).

Data analysis

To address potential selection bias as missing values may depend on some sociodemographic characteristics, multiple imputation by chained equations (MICE) was used.[24] Missing at random was assumed, as we found some variables were associated with missing observations. Using STATA (command: mi impute chained), 20 imputted datasets were produced. We improved the prediction by including baseline sample weights[25] and a set of variables available in the dataset were used as auxiliary variables[21] (i.e., not part of the main variables for the analysis, but are associated with any missing variable or predict missingness) in the model. Auxiliary variables

were main caregiver characteristics (psychological distress, longstanding illness, general health), child characteristics (longstanding illness, general health), family and neighbourhood-related characteristics (perception of financial status, housing tenure, number of people living in the household, and neighbourhood socioeconomic status) (as in [8]). We also included stratum from the study design as a predictor (e.g., as in [21]) in the model. Our final sample size after using MICE was the same as the maximum analytical sample size for different study baselines mentioned in the "Study design and data" subsection. We then set up the imputted datasets for complex survey design analysis (command: mi svyset).[25]

Supplementary tables

Table S1. Associations between indexes of psychosocial factors and the development of overweight and obesity to a follow-up of age 17 with additional adjustment for BMI z-score at baseline

**Psychosocial factors**	**Baseline: Age 11 (n = 7,979)**			**Baseline: Age 14 (n = 6,906)**		
	**Development (vs. no) by age 17**			**Development (vs. no) by age 17**		
	**OR**	**95% CI**	**p-value**	**OR**	**95% CI**	**p-value**
Child-reported psychosocial well-being	1.04	0.95, 1.15	0.382	1.08	0.97, 1.21	0.176
Caregiver-reported child mental health	1.15	1.02, 1.29	0.020	1.00	0.89, 1.11	0.943

**The p-value is statistically significant based on the Benjamini-Hochberg correction method (see Tables S9 and S10).*

OR = odds ratio; CI = confidence intervals

Indexes of psychosocial factors were presented in z-scores (mean = 0; SD = 1)

Both indexes were analysed in the same regression model, adjusting for sociodemographic covariates, pubertal status, and baseline BMI z-score.

Table S2. Interaction between indexes of psychosocial factors and index of socioeconomic status in predicting the development of overweight and obesity to a follow-up of age 17

**Psychosocial factors * SES**	**Baseline: Age 11 (n = 7,979)**						**Baseline: Age 14 (n = 6,906)**					
	**Development (vs. no) by age 17**			**Residualised change scores from ages 11 to 17**			**Development (vs. no) by age 17**			**Residualised change scores from ages 14 to 17**		
	**OR**	**95% CI**	**p-value**	**β**	**95% CI**	**p-value**	**OR**	**95% CI**	**p-value**	**β**	**95% CI**	**p-value**
Child-reported psychosocial well-being * SES	0.99	0.90, 1.09	0.861	-0.00	-0.04, 0.03	0.779	1.00	0.90, 1.11	0.973	-0.00	-0.03, 0.02	0.878
Caregiver-reported child mental health * SES	1.01	0.90, 1.12	0.903	0.03	-0.01, 0.07	0.172	1.00	0.91, 1.11	0.986	-0.00	-0.03, 0.02	0.854

**The p-value is statistically significant based on the Benjamini-Hochberg correction method (see Tables S9 and S10).*

OR = odds ratio; β = regression coefficient; CI = confidence intervals; SES = socioeconomic status

Indexes of psychosocial factors and socioeconomic status were presented in z-scores (mean = 0; SD = 1)

Interaction terms between indexes of psychosocial factors and index of socioeconomic status were analysed in the same regression model, adjusting for sociodemographic covariates (except for SES measures), indexes of psychosocial factors, and index of socioeconomic status.

Table S3. Associations between indexes of psychosocial factors at age 11 and the development of overweight and obesity by age 14

**Psychosocial factors**	**n = 8,004**					
	**Development (vs. no) by age 14**			**Residualised change scores from ages 11 to 14**		
	**OR**	**95% CI**	**p-value**	**β**	**95% CI**	**p-value**
Child-reported psychosocial well-being	0.97	0.88, 1.07	0.555	0.00	-0.02, 0.02	0.944
Caregiver-reported child mental health	1.25	1.11, 1.41	<0.001*	0.07	0.05, 0.09	<0.001*

**The p-value is statistically significant based on the Benjamini-Hochberg correction method (see Tables S9 and S10).*

OR = odds ratio; β = regression coefficient; CI = confidence intervals

Indexes of psychosocial factors were presented in z-scores (mean = 0; SD = 1)

Both indexes were analysed in the same regression model, adjusting for sociodemographic covariates and pubertal status.

Table S4. Interactions between baselines and indexes of psychosocial factors in predicting the development of overweight and obesity to a follow-up of age 17

**Reference:** **Psychosocial factors * Age 14**	**n = 14,885**					
	**Development (vs. no) by age 17**			**Residualised change scores from ages 11 or 14 to 17**		
	**OR**	**95% CI**	**p-value**	**β**	**95% CI**	**p-value**
Child-reported psychosocial well-being * Age 11	0.99	0.86, 1.14	0.852	0.03	-0.01, 0.07	0.175
Caregiver-reported child mental health * Age 11	1.08	0.95, 1.23	0.220	0.06	0.02, 0.11	0.003*

**The p-value is statistically significant based on the Benjamini-Hochberg correction method (see Tables S9 and S10).*

OR = odds ratio; β = regression coefficient; CI = confidence intervals

Indexes of psychosocial factors were presented in z-scores (mean = 0; SD = 1)

Both indexes were analysed in the same regression model, adjusting

Interaction terms between baselines (ages 11 vs. 14) and indexes of psychosocial factors were analysed in the same regression model, adjusting for sociodemographic covariates, pubertal status, baseline (ages 11 vs. 14), and indexes of psychosocial factors.

Table S5. Associations between individual psychosocial factors at age 11 and the development of overweight and obesity by age 14

**Psychosocial factors**	**n = 8,004**					
	**Development (vs. no) by age 14**			**Residualised change scores from age 11 to 14**		
	**OR**	**95% CI**	**p-value**	**β**	**95% CI**	**p-value**
Appearance satisfaction	0.98	0.89, 1.08	0.653	-0.01	-0.03, 0.01	0.496
Callous/unemotional traits	1.05	0.94, 1.17	0.389	0.01	-0.01, 0.03	0.453
Depressive symptoms	1.03	0.93, 1.13	0.596	0.01	-0.01, 0.03	0.267
Externalising symptoms	1.22	1.09, 1.37	0.001*	0.07	0.05, 0.09	<0.001*
Internalising symptoms	1.17	1.06, 1.28	0.001*	0.05	0.03, 0.07	<0.001*
Life satisfaction	1.01	0.93, 1.11	0.745	-0.02	-0.03, 0.00	0.124
Peer bullying	1.08	0.99, 1.18	0.103	0.04	0.02, 0.06	<0.001*
Self-esteem	1.01	0.93, 1.10	0.827	-0.01	-0.03, 0.01	0.444

**The p-value is statistically significant based on the Benjamini-Hochberg correction method (see Tables S9 and S10).*

OR = odds ratio; β = regression coefficient; CI = confidence intervals

All individual psychosocial factors were presented in z-scores (mean = 0; SD = 1)

Separate regression models were developed for each individual psychosocial factor, adjusting for sociodemographic covariates and pubertal status.

Table S6. Interactions between baselines and individual psychosocial factors in predicting the development of overweight and obesity to a follow-up of age 17

**Reference:**	**n = 14,885**					
**Psychosocial factor * Age 14**	**Development (vs. no) by age 17**			**Residualised change scores from ages 11 or 14 to 17**		
	**OR**	**95% CI**	**p-value**	**β**	**95% CI**	**p-value**
Appearance satisfaction * Age 11	1.02	0.89, 1.17	0.776	-0.03	-0.07, 0.01	0.100
Depressive symptoms * Age 11	1.02	0.89, 1.18	0.745	0.04	0.00, 0.07	0.028
Externalising symptoms * Age 11	1.09	0.95, 1.25	0.203	0.07	0.02, 0.11	0.002*
Internalising symptoms * Age 11	1.07	0.93, 1.22	0.330	0.06	0.02, 0.10	0.002*
Life satisfaction * Age 11	1.11	0.97, 1.27	0.140	-0.01	-0.05, 0.03	0.695
Peer bullying * Age 11	1.17	1.03, 1.33	0.014	0.07	0.04, 0.11	<0.001*
Self-esteem * Age 11	1.02	0.90, 1.16	0.713	-0.03	-0.07, 0.00	0.077

**The p-value is statistically significant based on the Benjamini-Hochberg correction method (see Tables S9 and S10).*

OR = odds ratio; β = regression coefficient; CI = confidence intervals

All individual psychosocial factors were presented in z-scores (mean = 0; SD = 1)

Separate regression models were developed for each individual psychosocial factor, adjusting for sociodemographic covariates, pubertal status, baseline (ages 11 vs. 14), and corresponding psychosocial factor.

Only individual psychosocial factors consistently available in both ages 11 and 14 were examined (n = 7).

Table S7. Associations between internalising, externalising, and peer bullying at age 11 and the development of overweight and obesity by age 17 fitted in the same regression model

Psychosocial factors	n = 7,979					
	**Development (vs. no) by age 17**			**Residualised change scores from age 11 to 17**		
	**OR**	**95% CI**	**p-value**	**β**	**95% CI**	**p-value**
Externalising symptoms	1.17	1.05, 1.30	0.006	0.09	0.05, 0.13	<0.001*
Internalising symptoms	0.99	0.90, 1.10	0.868	-0.01	-0.04, 0.03	0.749
Peer bullying	1.11	1.01, 1.22	0.033	0.05	0.02, 0.07	0.001*

**The p-value is statistically significant based on the Benjamini-Hochberg correction method (see Tables S9 and S10).*

OR = odds ratio; β = regression coefficient; CI = confidence intervals

All individual psychosocial factors were presented in z-scores (mean = 0; SD = 1)

Individual psychosocial factors (internalising symptoms, externalising symptoms, and peer bullying) were analysed in the same regression model, adjusting for sociodemographic covariates and pubertal status.

Table S8. Associations between internalising, externalising, and peer bullying at age 11 and the development of overweight and obesity by age 14 fitted in the same regression model

Psychosocial factors	n = 8,004					
	**Development (vs. no) by age 14**			**Residualised change scores from age 11 to 14**		
	**OR**	**95% CI**	**p-value**	**β**	**95% CI**	**p-value**
Externalising symptoms	1.16	1.03, 1.32	0.018	0.06	0.04, 0.08	<0.001*
Internalising symptoms	1.08	0.97, 1.20	0.156	0.02	-0.01, 0.04	0.203
Peer bullying	1.03	0.93, 1.13	0.613	0.02	0.00, 0.04	0.030

**The p-value is statistically significant based on the Benjamini-Hochberg correction method (see Tables S9 and S10).*

OR = odds ratio; β = regression coefficient; CI = confidence intervals

All individual psychosocial factors were presented in z-scores (mean = 0; SD = 1)

Individual psychosocial factors (internalising symptoms, externalising symptoms, and peer bullying) were analysed in the same regression model, adjusting for sociodemographic covariates and pubertal status.

Table S9. Benjamini-Hochberg adjustment method for the additional analyses of the associations between psychosocial factors and development (vs. no)

Development (vs. no)				
**Tables**	**Variables - baseline, follow-up**	**p-value**	**Rank**	**(i/n)q**
S3	Caregiver-reported child mental health - Age 11,14	<0.001	1	0.001
4	Externalising symptoms - Age 11,17	0.001	2	0.002
S5	Internalising symptoms - Age 11,14	0.001	3	0.003
S5	Externalising symptoms - Age 11,14	**0.001**	**4**	**0.004**
4	Peer bullying - Age 11,17	0.006	5	0.005
S7	Externalising symptoms - Age 11,17	0.006	6	0.006
4	Appearance satisfaction - Age 14,17	0.008	7	0.007
S6	Peer bullying - Interaction (11/14), 17	0.014	8	0.008
4	Self-esteem - Age 14,17	0.015	9	0.009
4	Online bullying - Age 14,17	0.017	10	0.010
S8	Externalising symptoms - Age 11,14	0.018	11	0.011
S1	Caregiver-reported child mental health - Age 11,17	0.020	12	0.012
S7	Peer bullying - Age 11,17	0.033	13	0.013
4	Appearance satisfaction - Age 11,17	0.050	14	0.014
4	Internalising symptoms - Age 11,17	0.060	15	0.015
4	Self-esteem - Age 11,17	0.098	16	0.016
S5	Peer bullying - Age 11,14	0.103	17	0.017
4	Depressive symptoms - Age 11,17	0.128	18	0.018
S6	Life satisfaction - Interaction (11/14), 17	0.140	19	0.019
4	Depressive symptoms - Age 14,17	0.154	20	0.020
S8	Internalising symptoms - Age 11,14	0.156	21	0.021
4	Life satisfaction - Age 14,17	0.162	22	0.022
S1	Child-reported psychosocial well-being- Age 14,17	0.176	23	0.023
S6	Externalising symptoms - Interaction (11/14), 17	0.203	24	0.024
S4	Caregiver-reported child mental health - Interaction (11/14), 17	0.220	25	0.025
S6	Internalising symptoms - Interaction (11/14), 17	0.330	26	0.026
S1	Child-reported psychosocial well-being- Age 11,17	0.382	27	0.027
S5	Callous/unemotional traits - Age 11,14	0.389	28	0.028
4	Life satisfaction - Age 11,17	0.417	29	0.029
4	Externalising symptoms - Age 14,17	0.486	30	0.030
S3	Child-reported psychosocial well-being- Age 11,14	0.555	31	0.031
S5	Depressive symptoms - Age 11,14	0.596	32	0.032
4	Peer bullying - Age 14,17	0.602	33	0.033
S8	Peer bullying - Age 11,14	0.613	34	0.034
S5	Appearance satisfaction - Age 11,14	0.653	35	0.035
S6	Self-esteem - Interaction (11/14), 17	0.713	36	0.036
4	Internalising symptoms - Age 14,17	0.725	37	0.037
S5	Life satisfaction - Age 11,14	0.745	38	0.038
S6	Depressive symptoms - Interaction (11/14), 17	0.745	39	0.039
S6	Appearance satisfaction - Interaction (11/14), 17	0.776	40	0.040
4	Social support - Age 14,17	0.800	41	0.041
S5	Self-esteem - Age 11,14	0.827	42	0.042

S4	Child-reported psychosocial well-being- Interaction (11/14), 17	0.852	43	0.043
S2	Child-reported psychosocial well-being- Age 11,17	0.861	44	0.044
S7	Internalising symptoms - Age 11,17	0.868	45	0.045
S2	Caregiver-reported child mental health - Age 11,17	0.903	46	0.046
4	Callous/unemotional traits - Age 11,17	0.904	47	0.047
S1	Caregiver-reported child mental health - Age 14,17	0.943	48	0.048
S2	Child-reported psychosocial well-being- Age 14,17	0.973	49	0.049
S2	Caregiver-reported child mental health - Age 14,17	0.986	50	0.050

i = the individual p-value's rank; n = total number of tests (50); q = the false discovery rate (5%)

The largest p-value lower than the Benjamini-Hochberg critical value is 0.001 (Rank 4). Therefore, p-values ≤ 0.001 are considered statistically significant.

Table S10. Benjamini-Hochberg adjustment method for the additional analyses of the associations between psychosocial factors and residualised change scores

Residualised changed scores				
**Tables**	**Variables - baseline, follow-up**	**p-value**	**Rank**	**(i/n)q**
4	Externalising symptoms - Age 11,17	<0.001	1	0.001
4	Peer bullying - Age 11,17	<0.001	2	0.002
S3	Caregiver-reported child mental health - Age 11,14	<0.001	3	0.003
S5	Internalising symptoms - Age 11,14	<0.001	4	0.004
S5	Externalising symptoms - Age 11,14	<0.001	5	0.005
S5	Peer bullying - Age 11,14	<0.001	6	0.007
S6	Peer bullying - Interaction (11/14), 17	<0.001	7	0.008
S7	Externalising symptoms - Age 11,17	<0.001	8	0.009
S8	Externalising symptoms - Age 11,14	<0.001	9	0.010
S7	Peer bullying - Age 11,17	0.001	10	0.011
S6	Internalising symptoms - Interaction (11/14), 17	0.002	11	0.012
S6	Externalising symptoms - Interaction (11/14), 17	0.002	12	0.013
S4	Caregiver-reported child mental health - Interaction (11/14), 17	0.003	13	0.014
4	Internalising symptoms - Age 11,17	**0.007**	**14**	**0.015**
S6	Depressive symptoms - Interaction (11/14), 17	0.028	15	0.016
S8	Peer bullying - Age 11,14	0.030	16	0.017
4	Depressive symptoms - Age 11,17	0.077	17	0.018
S6	Self-esteem - Interaction (11/14), 17	0.077	18	0.020
S6	Appearance satisfaction - Interaction (11/14), 17	0.100	19	0.021
S5	Life satisfaction - Age 11,14	0.124	20	0.022
4	Social support - Age 14,17	0.156	21	0.023
S2	Caregiver-reported child mental health - Age 11,17	0.172	22	0.024
S4	Child-reported psychosocial well-being- Interaction (11/14), 17	0.175	23	0.025
S8	Internalising symptoms - Age 11,14	0.203	24	0.026
4	Online bullying - Age 14,17	0.264	25	0.027
S5	Depressive symptoms - Age 11,14	0.267	26	0.028
4	Internalising symptoms - Age 14,17	0.289	27	0.029
4	Peer bullying - Age 14,17	0.322	28	0.030
4	Self-esteem - Age 11,17	0.378	29	0.032
4	Appearance satisfaction - Age 11,17	0.426	30	0.033
S5	Self-esteem - Age 11,14	0.444	31	0.034
S5	Callous/unemotional traits - Age 11,14	0.453	32	0.035
4	Externalising symptoms - Age 14,17	0.477	33	0.036
4	Callous/unemotional traits - Age 11,17	0.485	34	0.037
S5	Appearance satisfaction - Age 11,14	0.496	35	0.038
4	Life satisfaction - Age 14,17	0.547	36	0.039
S6	Life satisfaction - Interaction (11/14), 17	0.695	37	0.040
4	Depressive symptoms - Age 14,17	0.732	38	0.041
S7	Internalising symptoms - Age 11,17	0.749	39	0.042
4	Life satisfaction - Age 11,17	0.758	40	0.043
S2	Child-reported psychosocial well-being- Age 11,17	0.779	41	0.045
S2	Caregiver-reported child mental health - Age 14,17	0.854	42	0.046

4	Self-esteem - Age 14,17	0.862	43	0.047
S2	Child-reported psychosocial well-being- Age 14,17	0.878	44	0.048
4	Appearance satisfaction - Age 14,17	0.920	45	0.049
S3	Child-reported psychosocial well-being- Age 11,14	0.944	46	0.050

i = the individual p-value's rank; n = total number of tests (46); q = the false discovery rate (5%)

The largest p-value lower than the Benjamini-Hochberg critical value is 0.007 (Rank 14). Therefore, the findings with p-value ≤ 0.007 are considered statistically significant.

References

1 Kelly, Y., Patalay, P., Montgomery, S. & Sacker, A. Bmi Development and Early Adolescent Psychosocial Well-Being: Uk Millennium Cohort Study. *Pediatrics* **138** (2016).

2 Wright, C. M. et al. Growth Reference Charts for Use in the United Kingdom. *Arch Dis Child* **86**, 11-14 (2002).

3 Vidmar, S. I., Cole, T. J. & Pan, H. Standardizing Anthropometric Measures in Children and Adolescents with Functions for Egen: Update. *The Stata Journal* **13**, 366-378 (2013).

4 Hudda, M. T. et al. Patterns of Childhood Body Mass Index (Bmi), Overweight and Obesity in South Asian and Black Participants in the English National Child Measurement Programme: Effect of Applying Bmi Adjustments Standardising for Ethnic Differences in Bmi-Body Fatness Associations. *Int J Obes (Lond)* **42**, 662-670 (2018).

5 Centre for Longitudinal Studies. *Child Overweight and Obesity: Initial Findings from the Millennium Cohort Study Age 14 Survey*, <https://cls.ucl.ac.uk/wp-content/uploads/2017/12/MCS6-Briefing-02-Overweight-and-obesity.pdf> (2017).

6 Goodman, R. The Strengths and Difficulties Questionnaire: A Research Note. *J Child Psychol Psychiatry* **38**, 581-586 (1997).

7 Papachristou, E. & Flouri, E. Distinct Developmental Trajectories of Internalising and Externalising Symptoms in Childhood: Links with Mental Health and Risky Behaviours in Early Adolescence. *Journal of Affective Disorders* **276**, 1052-1060 (2020).

8 Putra, I. G. N. E., Daly, M. & Robinson, E. Psychological Well-Being and the Reversal of Childhood Overweight and Obesity in the Uk: A Longitudinal National Cohort Study. *Obesity* (2024).

9 Rosenberg, M. *Society and the Adolescent Self-Image* (Princeton University Press, 1965).

10 Bannink, R., Pearce, A. & Hope, S. Family Income and Young Adolescents' Perceived Social Position: Associations with Self-Esteem and Life Satisfaction in the Uk Millennium Cohort Study. *Arch Dis Child* **101**, 917-921 (2016).

11 Creese, H., Saxena, S., Nicholls, D., Pascual Sanchez, A. & Hargreaves, D. The Role of Dieting, Happiness with Appearance, Self-Esteem, and Bullying in the Relationship between Mental Health and Body-Mass Index among Uk Adolescents: A Longitudinal Analysis of the Millennium Cohort Study. *eClinicalMedicine* **60** (2023).

12 Essau, C. A., Sasagawa, S. & Frick, P. J. Callous-Unemotional Traits in a Community Sample of Adolescents. *Assessment* **13**, 454-469 (2006).

13 Sharpe, H., Fink, E., Duffy, F. & Patalay, P. Changes in Peer and Sibling Victimization in Early Adolescence: Longitudinal Associations with Multiple Indices of Mental Health in a Prospective Birth Cohort Study. *Eur Child Adolesc Psychiatry* **31**, 737-746 (2022).

14 Angold, A., Costello, E. J., Messer, S. C. & Pickles, A. Development of a Short Questionnaire for Use in Epidemiological Studies of Depression in Children and Adolescents. *International Journal of Methods in Psychiatric Research* **5**, 237-249 (1995).

15 Booker, C. L., Skew, A. J., Sacker, A. & Kelly, Y. J. Well-Being in Adolescence—an Association with Health-Related Behaviors: Findings from Understanding Society, the Uk Household Longitudinal Study. *The Journal of Early Adolescence* **34**, 518-538 (2013).

16 Yang, K., Petersen, K. J. & Qualter, P. Undesirable Social Relations as Risk Factors for Loneliness among 14-Year-Olds in the Uk: Findings from the Millennium Cohort Study. *International Journal of Behavioral Development* **46**, 3-9 (2020).

17 Cutrona, C. E. & Russell, D. W. The Provisions of Social Relationships and Adaptation to Stress. *Advances in Personal Relationships* **1**, 37-67 (1987).
18 Fenn, J., Tan, C.-S. & George, S. Development, Validation and Translation of Psychological Tests. *BJPsych Advances* **26**, 306-315 (2020).
19 Osborne, J. W. What Is Rotating in Exploratory Factor Analysis? *Practical Assessment, Research, and Evaluation* **20** (2015).
20 Sacre, H. et al. Development and Validation of the Socioeconomic Status Composite Scale (Ses-C). *BMC Public Health* **23**, 1619 (2023).
21 Staatz, C. B., Kelly, Y., Lacey, R. E. & Hardy, R. Area-Level and Family-Level Socioeconomic Position and Body Composition Trajectories: Longitudinal Analysis of the Uk Millennium Cohort Study. *The Lancet Public Health* **6**, e598-e607 (2021).
22 Hazell, M., Thornton, E., Haghparast-Bidgoli, H. & Patalay, P. Socio-Economic Inequalities in Adolescent Mental Health in the Uk: Multiple Socio-Economic Indicators and Reporter Effects. *SSM - Mental Health* **2**, 100176 (2022).
23 Pongiglione, B. & Fitzsimons, E. Overweight and Obesity in Childhood and Adolescence: Findings from the Uk Millennium Cohort Study, up to Age 14. *Longitudinal and Life Course Studies* **10**, 27-50 (2019).
24 Azur, M. J., Stuart, E. A., Frangakis, C. & Leaf, P. J. Multiple Imputation by Chained Equations: What Is It and How Does It Work? *Int J Methods Psychiatr Res* **20**, 40-49 (2011).
25 Manly, C. A. & Wells, R. S. Reporting the Use of Multiple Imputation for Missing Data in Higher Education Research. *Research in Higher Education* **56**, 397-409 (2015).
